# microRNAs: short non-coding bullets of gain of function mutant p53 proteins

**DOI:** 10.18632/oncoscience.52

**Published:** 2014-06-07

**Authors:** Sara Donzelli, Sabrina Strano, Giovanni Blandino

**Affiliations:** ^1^ Translational Oncogenomic Lab, Italian National Cancer Institute “Regina Elena”, Rome, Italy; ^2^ Molecular Chemoprevention Group, Italian National Cancer Institute “Regina Elena”, Rome, Italy

**Keywords:** mutant p53, microRNAs, gain of function

## Abstract

TP53 gene mutations are present in more than half of all human cancers. The resulting proteins are mostly full-length with a single aminoacid change and are abundantly present in cancer cells. Some of mutant p53 proteins gain oncogenic activities through which actively contribute to the aberrant cell proliferation, increased resistance to apoptotic stimuli and ability to metastatize of cancer cells. Gain of function mutant p53 proteins can transcriptionally regulate the expression of a large plethora of target genes. This mainly occurs through the formation of oncogenic transcriptional competent complexes that include mutant p53 protein, known transcription factors, posttranslational modifiers and scaffold proteins. Mutant p53 protein can also transcriptionally regulate the expression of microRNAs, small non-coding RNAs that regulate gene expression at the posttranscriptional level. Each microRNA can putatively target the expression of hundred mRNAs and consequently impact on many cellular functions. Thus, gain of function mutant p53 proteins can exert their oncogenic activities through the modulation of both non-coding and coding regions of human genome.

Research effort in the last two decades revealed transcriptional activity within non-coding and coding regions of the entire human genome. This led to the identification of non-coding transcripts that are mainly located in the cell nucleus and expressed at lower levels than coding-RNAs [1-4]. The lack of functional annotations made the classification of the different non-coding RNA populations rather difficult. Indeed, an arbitrary cut-off based on the length of non-coding RNAs distinguished long non-coding RNAs (lncRNAs, 100-200nt) from microRNAs (miRNAs), small interfering RNAs (siRNAs), and Piwi-interacting RNAs (piRNAs) that span 21-35 nucleotides respectively [5]. To date, microRNAs are those non-coding RNAs that were mostly studied and closely linked to human cancers [6-9]. MicroRNAs are evolutionarily conserved small non-coding RNAs that regulate gene expression at the posttranscriptional level. This occurs through imperfect complementarity to the 3′untranslated region (3′UTR) of target mRNAs which results in mRNAs translational inhibition and/or degradation and leads ultimately to a reduction in protein expression level. MicroRNAs are predicted to target over 50% of all human protein-coding genes and each gene could be controlled by different microRNAs [10]. Thus, many if not all, cellular functions can be putatively subjected to microRNA control. MicroRNAs were originally identified as regulators of developmental processes including developmental timing and cell fate transitions [11, 12]. Croce’s group originally reported the involvement of microRNAs in chronic lymphocytic leukemia (CLL) [13]. Many miRNAs map to specific regions of the human genome frequently deleted or amplified in human cancers [14-16]. Growing evidence has shown that miRNAs might be differentially expressed in cancer cells, in which they form unique expression patterns or signatures [7]. Altered expression of miRNAs in cancers can occur through epigenetic changes, including aberrant DNA methylation and histone modifications, aberrant transcriptional regulation and genetic alterations [17, 18]. These alterations can affect the production of the primary RNAs, their processing to the mature miRNA forms, and/or interactions with mRNA targets. Mutations in the genes encoding for proteins involved in the processing and maturation of miRNAs such as TARBP2, DICER1 and XPO5 have been found to lead to overall reductions in miRNA expression [19-21]. miRNAs can either act as tumor suppressor genes or oncogenes [22]. Increasing evidence indicate that the expression of miRNAs is mainly downregulated in tumor tissues, as compared to corresponding healthy tissues, which might suggest that miRNAs are primarily tumor suppressor genes [23, 24]. Extensive down-regulation of multiple miRNAs followed rapidly EGF stimulation of breast cancer cells [23]; this was paired by the induction of target mRNAs with oncogenic activities [23]. Several well-characterized oncogenic miRNAs were in tumors. The expression of miRNA 21-5p is very frequently up-regulated in diverse tumoral tissues when compared to both matched or unmatched non-tumoral ones [25, 26]. miRNA 21-5p overexpression promotes tumor growth and invasion [27-30]. Ectopic expression of miR-7 promotes cell growth and tumor formation in lung cancer cells [31]. EGFR activation induces miR-7 expression through a RAS-MYC pathway. Indeed MYC binds to and activates the miR-7 promoter. Aberrant transcriptional regulation of miRNA-10b5p by Twist promotes breast cancer metastatization [32]. Exposure to both metabolic cancer risk factors and to carcinogenic substances such as asbestos, formaldehyde and cigarette smoke in lung and hepatic tissues alters miRNA expression[33-37]. This might unveil miRNAs as both predictors and players of cancer development.

P53 gene is the most frequent target for genetic alterations in human cancers [38]. Indeed, more than half of human cancers carry p53 mutations. Most of these mutations are missense and reside in the core domain of p53 protein [39]. They can be roughly divided in two main classes: (a) *DNA contact defective* mutants whose residue subjected to mutation is located in the region of the protein that binds to DNA; (b) *Structural defective* mutants whose mutation impinges on a residue critical for the entire folding of the protein [40]. There are two main features that distinguish wt-p53 and mutant p53 proteins. Mutant p53 proteins are unable to bind to wt-p53 DNA binding consensus and consequently are unable to promote transcriptional dependent wt-p53 tumor suppressor activities. Mutant p53 are rather stable proteins as their half-life is extremely prolonged when compared to that of wt-p53 protein. While in the past decades mutant p53 proteins were mainly considered as loss of function gene products with no specific activities, increasing evidence have established that these proteins gain additional functions through which strongly contribute the transformed phenotype of a given tumor cell [41-43]. Since mutant p53 proteins are abundantly present in many human cancers, the rationale to envisage them as an important target for novel cancer therapeutic intervention is very strong and consequently is arousing a remarkable interest. This also led to extraordinary experimental effort to decipher the molecular mechanisms underlying the oncogenic activities of mutant p53 proteins (Figure 1). Mutant p53 proteins can aberrantly modulate the expression of genes acting as oncogenic transcription factors. Di Agostino et al., originally reported that mutant p53 physically interacted with the transcription factor NF-Y [44]. This led to the transcriptional upregulation of the expression of cell cycle regulated genes such as cyclin B, cdk1 and cdc25. Notably, mutant p53 proteins were recruited onto the DNA binding consensus for the transcription factor NF-Y, thus implying its direct involvement on transcriptional machinery that leads to the aberrant regulation of NF-Y target genes. Since these observations, other transcriptional crosstalks involving other transcription factors such as VDR, SP1, E2F1, ETS1, NFKb, were reported [45-49]. These findings implicate that gain of function mutant p53 proteins can broadly modulate gene expression as they can parasite the activity of the interacting transcription factors. Mutant p53 proteins could putatively modulate the expression of most, if not all, gene targets of the parasite transcription factors. There is still very scarce evidence on which is the role of mutant p53 proteins in the context of these oncogenic transcriptional competent complexes. It was originally reported that mutant p53 protein in complex with the transcription factor NFY favored the recruitment of the acetylase p300; thereby potentiating the activity of the interacting transcription factor [44]. Gain of function mutant p53 proteins disabled the transcriptional repressor complex p73/NFY assembled on PDGFr promoter. This led to pancreatic cancer metastasis [50]. In advanced cancer lesions TFGβ ligands act as pro-metastatic factors. Adorno et al., reported that oncogenic Ras and mutant p53 favored the assembling of a protein complex mutant p53/p63 via Smad proteins [51]. This antagonized p63 tumor suppressor activities by impairing its transcriptional function [51]. Mutant p53 and p300 were co-recruited at the promoter of REGg, a proteasome activator, which is overexpressed in tumors and promotes degradation of p53, p21 and p16 [52]. In that context, mutant p53 also prevented the formation of Smad3/N-CoR complex on the REGg promoter and thereby enhancing the activity of the REGg-proteasome within gain of function of mutant p53 proteins. It was recently reported that mutant p53 protein transcriptionally activates SREBP gene and through it aberrantly regulates the mevalonate pathway [53]. Notably, increased levels of mevalonate by mutant p53 proteins promoted nuclear localization and oncogenic activation of two nodal transducers of HIPPO tumor suppressor pathway, named YAP and TAZ [54]. Additional partners can be present in the mutant p53 transcriptional competent complexes and potentiate its activity. The prolyl-isomerase PIN1 enhanced mutant p53 transcriptional activity and conferred gene target specificity in breast cancer cells [55].

**Figure 1:**
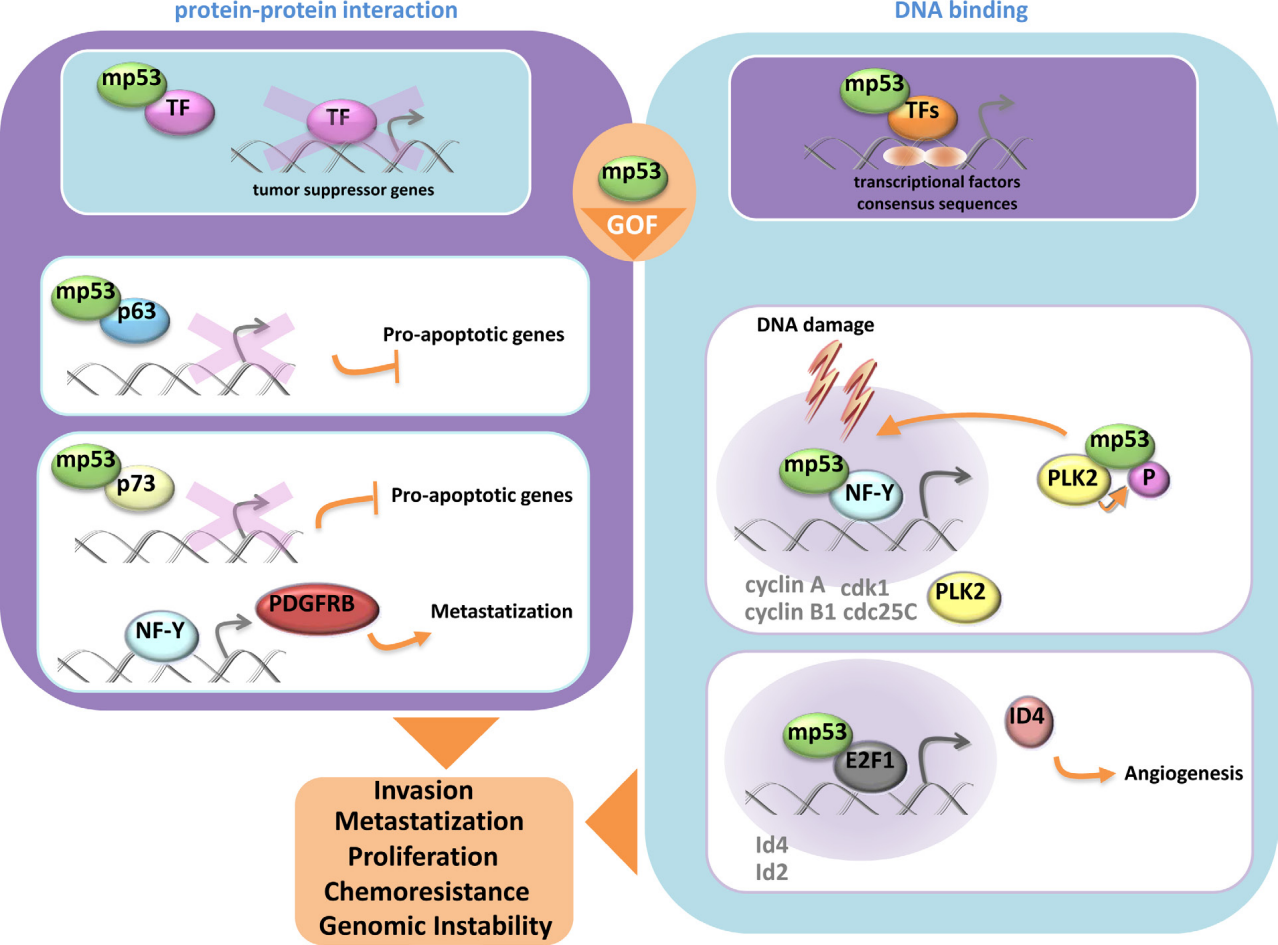
Mutant p53protein gain of function activity

Donzelli et al., originally reported that gain of function mutant p53 proteins can also modulate the expression of microRNAs [56]. Mutant p53 proteins were found on the promoter of ARPP21, the host gene of the oncogenic miR-128b. Aberrant expression of miR-128b contributed to increase chemoresistance of lung cancer cells by targeting the transcriptional repressor E2F5 [56]. This led to increased cytoplasmic accumulation of p21 that in turn reduced the apoptotic rate of lung cancer cells to commonly used anticancer treatments. Masciarelli et al demonstrated that gain of function mutant p53 proteins transcriptionally down-regulated the expression of miR-223 [57]. This resulted in the augmented expression of its mRNA target stathmin-1, an oncoprotein known to confer increased chemoresistance and associated with poor clinical prognosis [57]. Interestingly, a transcriptional cross-talk with mutant p53 and the transcriptional repressor ZEB1, occurring on the regulatory regions of miR-223, was also documented [57]. A direct involvement of gain of function mutant p53 proteins in the processing of microRNAs has been recently evidenced. Mutant p53 proteins downregulated Dicer expression thereby conferring to cancer cells a more invasive and metastatic phenotype as for Dicer depletion [58]. It was recently reported that mutant could promote epithelial-mesenchymal transition (EMT) and tumor metastasis by transcriptionally inhibiting miR-130 that has among its mRNA target the transcriptional repressor ZEB1 [59]. Reduced expression of miR-130 released ZEB1-mediated EMT [59]. Mutant p53-induced up-regulation of miR-155 favored invasion of breast cancer cells [60]. MicroRNA-let7i was shown to be transcriptionally downregulated by diverse mutant p53 proteins. This led to increased invasion and migration of breast cancer cells [61]. MicroRNA-27 was reported to be transcriptionally downregulated by mutant p53 protein [62]. This released aberrant expression of its mRNA target, EGFR, which consequently instigated an increased and prolonged signaling promoting tumor development [62]. The tumor spectrum on both p53 null mice and p53 His172 knock-in mice is similar, but the tumors developed by the latter gain the ability to metastatize. The transcriptional activity of mutant p53 proteins as the ability to modulate both coding and non-coding RNAs appears to play a critical role in mutant p53-mediated tumorigenesis (figure 2). Many studies have reported that patients with tumors carrying p53 mutations have worse prognosis, shorter survival and poorer response to conventional anticancer treatments that those bearing wt-p53 protein [43, 63]. P53 mutations frequently associated with aberrant, either up- or down-regulated, expression of their transcriptional target genes [64]. Ganci et al., recently reported that TP53 mutations associated with a shorter recurrence-free survival of head and neck patients [65]. The expression pattern of 49 miRs associated with TP53 status. In particular, within the 49 miRs, a group of 12 miRs correlated with recurrence free-survival and a group of 4 miRs with cancer-specific survival [65]. Altogether these findings indicate that specific microRNA expression associated with TP53 missense mutations and with reduced survival after surgical treatment of patients with head and neck squamous cell carcimonas.

**Figure 2:**
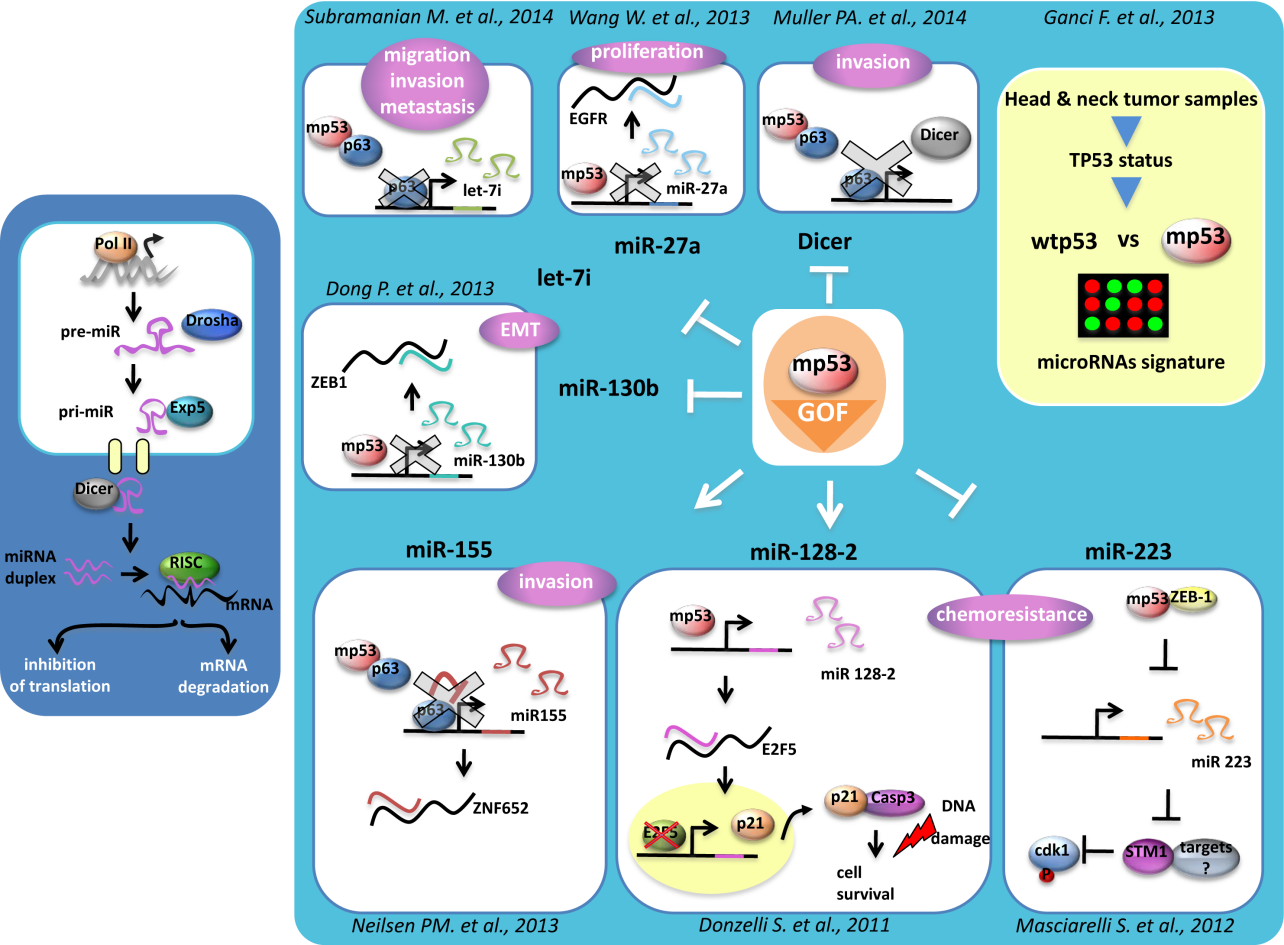
MicroRNAs regulation by mutant p53 protein

Most of the existing evidence for p53 mutations is related to missense mutations, but other type of mutations, such as non-sense and frameshifts are also present in human cancers. The understanding of their contribution to cancer phenotype will provide a comprehensive view of the role of TP53 alterations in human cancers. This also needs to be referred to the presence of the p53 family members p73 and p63 and its derived isoforms that compose a network of more than 20 polypeptides floating in cancer cells. It was originally shown that mutant p53 proteins can physically associate with either p73 or p63 and through it impaired p73- or p63 transcriptional activation and consequently their antitumoral activities [66-69]. Small interfering peptides disassembling the oncogenic protein complex mutp53/ p73 restored p73 oncosuppressor activities [70, 71]. This leads to propose that protein/protein interactions are critical for the oncogenic activities of mutant p53 proteins either to parasite or to inhibit the activity of transcription factors. In such a functional context, the core domain of mutant p53, where most of mutations reside, plays an important role as an interacting platform. Since the mutation of a single p53 residue impacts differently on the overall structure of the protein it can be hypothesized that diverse interacting platforms as for the diverse p53 mutations are present in human cancers. Little is known about the determinants that dictate which are the preferential interacting proteins of a given mutant p53 platform. This may impart specificity in the selection of which transcriptional target will be modulated by gain of function mutant p53 protein. For instance, the inability of mutant p53 proteins to transcriptionally active Mdm2 gene 431 has a profound impact on their half-life. Unlike wt-p53 protein that is extensively subjected to E3 ligase activity of MDM2, mutant p53 proteins evade such a tight control and consequently become rather stable and abundant in cancer cells [72, 73]. This might also occur for other negative regulators that cannot target mutant p53 proteins either because their binding affinity is lower than that to wt-p53 protein or mutant p53 is hidden in “protective” complexes with chaperons thereby making it unavailable to the degradation machinery [74]. It was also reported that mutant p53 proteins are unstable in normal tissues; thereby implying a critical role of the transformed cell context in the stabilization and oncogenic activities of mutant p53 proteins. The contribution of the transformed cell context to the oncogenic activity of mutant p53 proteins could also depend from the organ site of the tumor and from the specificity of the additional genetic alterations that occur in a given tumors. The full comprehension of the molecular events underlying gain of function of mutant p53 proteins is essential for improving: (a) the ongoing therapeutic approaches tackling mutant p53 gain of function; (b) the design of novel mutant p53 personalized cancer therapeutic approaches.
